# Increased metastasis with loss of *E2F2* in *Myc*-driven tumors

**DOI:** 10.18632/oncotarget.5690

**Published:** 2015-10-13

**Authors:** Inez Yuwanita, Danielle Barnes, Michael D. Monterey, Sandra O'Reilly, Eran R. Andrechek

**Affiliations:** ^1^ Department of Physiology, Michigan State University, East Lansing, MI48824, Michigan, USA

**Keywords:** MMTV-*Myc*, metastasis, E2F transcription factors, PTPRD, gene expression

## Abstract

In human breast cancer, mortality is associated with metastasis to distant sites. Therefore, it is critical to elucidate the biological mechanisms that underlie tumor progression and metastasis. Using signaling pathway signatures we previously predicted a role for E2F transcription factors in *Myc* induced tumors. To test this role we interbred MMTV-*Myc* transgenic mice with E2F knockouts. Surprisingly, we observed that the loss of *E2F2* sharply increased the percentage of lung metastasis in MMTV-*Myc* transgenic mice. Examining the gene expression profile from these tumors, we identified genetic components that were potentially involved in mediating metastasis. These genes were filtered to uncover the genes involved in metastasis that also impacted distant metastasis free survival in human breast cancer. In order to elucidate the mechanism by which *E2F2* loss enhanced metastasis we generated knockdowns of *E2F2* in MDA-MB-231 cells and observed increased migration *in vitro* and increased lung colonization *in vivo*. We then examined genes that were differentially regulated between tumors from MMTV-*Myc*, MMTV-*Myc E2F2*^−/−^, and lung metastases samples and identified *PTPRD*. To test the role of *PTPRD* in *E2F2*-mediated breast cancer metastasis, we generated a knockdown of *PTPRD* in MDA-MB-231 cells. We noted that decreased levels of *PTPRD* resulted in decreased migration *in vitro* and decreased lung colonization *in vivo*. Taken together, these data indicate that *E2F2* loss results in increased metastasis in breast cancer, potentially functioning through a *PTPRD* dependent mechanism.

## INTRODUCTION

Breast cancer has been shown to be a highly heterogeneous disease through genomic analysis of both human and the mouse tumor samples [1–7]. Classification based on gene expression profiles of tumor samples resulted in a detailed analysis of human breast cancer, allowing for a better understanding of the disease at both molecular and clinical levels. However, breast cancer is still lethal, predominately due to metastatic progression [8]. Breast cancer metastasis is a complex multistep process that involves detachment of tumor cells from the original site, intravasation into the blood vessel, extravasation out of the blood vessel, and colonization of distant organs such as the bone, brain, lung, and liver [9–11].

To study breast cancer development and progression, one approach that has been used is the generation of genetically engineered mouse model systems. One well studied model is the highly metastatic MMTV-PyMT strain, where metastasis to the lung is observed in virtually all mice reaching tumor endpoint [12, 13]. Conversely, the MMTV-*Myc* model [14] is poorly metastatic [15–17]. This suggests that MMTV-*Myc* tumors would require additional genetic events in order to model metastasis found in *c-Myc* associated human breast cancer metastasis.

In mouse models of breast cancer, the E2F transcription factors have been predicted to be activated in many tumor models [7]. Traditionally, E2F transcription factors have been well described to regulate cell cycle [18–20]. In cancer, E2Fs have been implicated in tumor development, progression [21] and angiogenesis [22]. Specifically in breast cancer, *E2F1* expression has been shown to be reduced in primary and metastatic breast carcinoma [23, 24] and deletion of the *E2F2* chromosomal region was also observed [25]. In the analysis of mouse mammary tumors from MMTV-*Myc* mice, an enrichment of E2F bound genes in the EMT/squamous subset of tumor samples was noted [26]. Recently, perturbation of individual E2Fs in the MMTV-PyMT model was shown to affect latency, histology, vasculature, and importantly, the metastatic capability of these tumors [13]. Specifically, *E2F1* and *E2F2* loss was shown to vastly reduce metastasis. Other studies have implicated the E2Fs in the tumor process, including an examination of *Neu* and *Myc* initiated tumors in the absence of various E2Fs with impacts on latency and metastasis that varied by model [27–29]. In the metastatic process, E2Fs have been shown to mediate metastasis by regulating matrix metalloproteinase [30] and that expression of *E2F1* increased extravasation of circulating cancer cells from the endothelium [31].

We have previously shown that the perturbation of activator E2Fs levels in the MMTV-*Myc* mouse model of breast cancer affected incidence and latency [28]. Here we demonstrate that loss of *E2F2* in *Myc* induced tumors dramatically increased breast cancer metastasis. To define the role of *E2F2* in the metastasis process, we generated gene expression data with and without *E2F2* in *Myc* induced tumors. Candidate genes regulating metastasis were identified and tested for roles in metastasis. This analysis demonstrated that a tumor suppressor gene, *PTPRD* [32–34], may act in conjunction with *E2F2* to mediate metastasis.

## RESULTS

### *E2F2* loss induces metastasis in MMTV-*Myc* driven tumors

**Figure 1:**
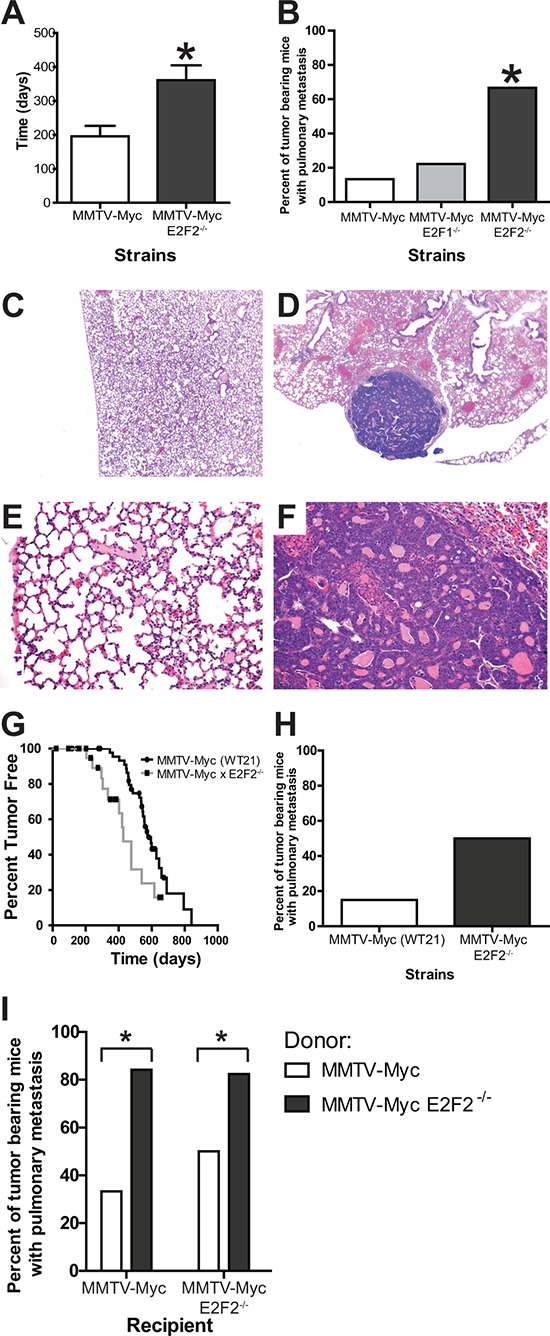
*E2F2* loss induces metastasis in *Myc* driven tumors MMTV-*Myc* transgenic mice were interbred with *E2F2*^−/−^ mice and tumor latency was examined. *Myc* tumors developing in the absence of *E2F2* had a significantly increased time to tumor onset (**A.** *p* = 0.0057). Metastasis is rarely observed in MMTV-*Myc* mice with only 13% of tumor bearing mice having lung metastasis (**B.** *n* = 2/15). Metastatic incidence is increased to 67% when *Myc* tumors develop in the *E2F2* knockout background (*n* = 6/9; *p*-value = 0.0361). Histology of a MMTV-*Myc* mouse lung showing the absence of lung metastasis at 4X **C.** compared with the metastases observed in the MMTV-*Myc E2F2* null strain **D.** Increased magnification (20X) of these sections revealed secondary structure within the metastatic lesion **E** and **F.** To ensure that the metastatic effect of *E2F2* loss was not a strain specific effect, MMTV-*Myc* WT21 mice were interbred with *E2F2*^−/−^ mice. Loss of *E2F2* in the MMTV-*Myc* WT21 background resulted in decreased latency **G.** and trend towards increased percentage of metastasis bearing mice **H.** Transplantation of MMTV-*Myc* WT21 *E2F2*^−/−^ tumors into MMTV-*Myc* WT21 or MMTV-*Myc* WT21 *E2F2*^−/−^ backgrounds produced striking metastases, suggesting that loss of *E2F2* affected metastasis in a cell autonomous manner **I.**

Our previous research had predicted and demonstrated a role for E2F pathway activation in MMTV-*Myc* tumors [28]. In that work, a Kaplan-Meier survival plot revealed a significant acceleration in tumor onset when *E2F1* was lost and a delay in tumor onset when *E2F2* or *E2F3* was lost. Indeed, we saw that loss of *E2F2* increased time to tumor onset by an average of 160 days (Figure 1A; *p* = 0.0057). Here we observed that the loss of any E2F increased metastases in MMTV-*Myc* intiated tumors which are normally not highly metastatic (13%). However, only the loss of *E2F2* significantly increased the percentage of tumor bearing mice with metastasis to the lung to 67% (Figure 1B; *p* = 0.0361).

Other published work noted that *E2F2* loss in Wap-*Myc* mice decreased time to tumor onset and did not describe a metastatic phenotype [29]. Given the differences in latency effects between the two published MMTV-*Myc* E2F reports, we sought to ensure that the metastatic effects were not a strain specific artifact. To do this, we interbred a separate MMTV-*Myc* transgenic, MMTV-*Myc* WT21 [26] with the *E2F2* knockouts. With the loss of *E2F2* in this strain, tumors developed 147 days earlier (Figure 1G). Importantly, despite the differences in latency, loss of *E2F2* in this strain also increased the metastatic frequency by 35% (Figure 1H), demonstrating the role of *E2F2* in MMTV-*Myc* mediated metastasis was not a strain dependent artifact. Furthermore, transplantation of MMTV-*Myc* WT21 *E2F2*^−/−^ tumors into MMTV-*Myc* WT21 or MMTV-*Myc* WT21 *E2F2*^−/−^ background revealed that loss of *E2F2* increased metastasis in a cell autonomous manner (Figure 1I; *p* = 0.0177 and 0.0382, respectively) which further confirmed our finding of the role of *E2F2* in MMTV-*Myc* mediated metastasis.

Histology of pulmonary sections for the MMTV-*Myc* strains typically resulted in sections lacking metastases (Figure 1C) in 87% of tumor bearing mice. Conversely, metastatic lesions were readily visible in the MMTV-*Myc* mice lacking *E2F2* (Figure 1D) in 67% of tumor bearing mice. At high power, normal lung morphology was observed in the MMTV-*Myc* strain (Figure 1E) while secondary structure in the lung metastases was observed (Figure 1F). Lung metastases at necropsy were occasionally large enough (2–3 mm in diameter) that flash frozen samples were isolated for gene expression studies.

### Gene expression alterations associated with lung metastasis

**Figure 2:**
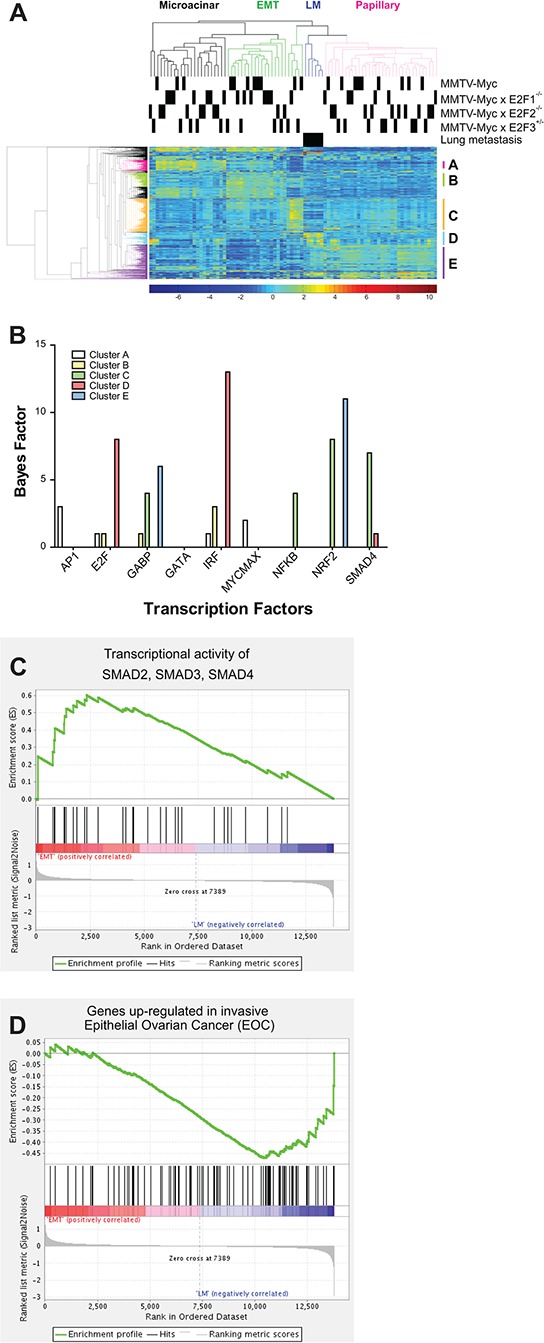
Gene expression alterations associated with lung metastasis Gene expression analysis of MMTV-*Myc* tumors in E2F the WT, *E2F1*^−/−^, *E2F2*^−/−^ and E2F3^+/−^ backgrounds and 6 sample of lung metastasis were analyzed by microarray. Unsupervised hierarchical clustering revealed that samples were clustered based on their histological type rather than their genotype with lung metastasis samples being clustered together **A.** The clustering of the metastatic samples in one group, closely related to the papillary subtype was noted. We examined the indicated sub-clusters of genes for predicted transcription factor binding demonstrating that genes upregulated in metastasis, represented in cluster D, were enriched for E2F binding motifs **B.** Comparison of EMT and lung metastasis samples by GSEA demonstrated enrichment of genes regulated by *SMAD2, SMAD3*, and *SMAD4* was elevated in EMT relative to lung metastases (**C.** *p* = 0.03). Enrichment of genes up-regulated in invasive ovarian epithelial cancer was noted in the metastasis samples (**D.** *p* = 0.016).

To begin to determine the mechanism by which loss of *E2F2* function increased metastasis in *Myc* induced tumors, we examined gene expression by microarray in MMTV-*Myc* and MMTV-*Myc E2F2*^−/−^ tumors. We included tumors from MMTV-*Myc E2F1*^−/−^ and MMTV-*Myc E2F3^+/−^* as controls for tumors with loss of activator E2Fs without the presence of metastases. In addition to 20 primary tumors from each genotype, we assayed 6 E2F mutant lung metastasis samples. Unsupervised hierarchical clustering divided these primary samples based on their histological type, rather than by their genotype. Interestingly, the lung metastasis samples clustered together and were most closely related to the papillary subtype. (Figure 2A). Focusing on metastasis in a clustering analysis, groups of genes that were upregulated uniquely in each cluster were identified. For instance, in Cluster D, 108 genes defined the 6 lung metastases samples and clearly differentiated between these samples and other samples. The transcriptional control of these groups was examined through an over-representation analysis using predicted transcription factor binding sites. This revealed an enrichment in genes with predicted E2F binding sites in genes upregulated in lung metastasis (gene cluster D; Figure 2B). Given that epithelial-mesenchymal transition (EMT) has been linked to the propensity of cells to metastasize [35], we compared EMT and lung metastasis clusters using GSEA. As expected, enrichment of genes regulated by *SMAD2, SMAD3*, and *SMAD4* were enriched in the EMT cluster (Figure 2C; *p* = 0.03). Interestingly, this experiment demonstrated an enrichment of an invasive ovarian epithelial cancer geneset in the lung metastastic samples, suggesting that similar gene expression patterns could be shared (Figure 2D, *p* = 0.016).

### Pipeline for identification of genes regulating *E2F2*^−/−^ breast cancer metastasis

**Figure 3:**
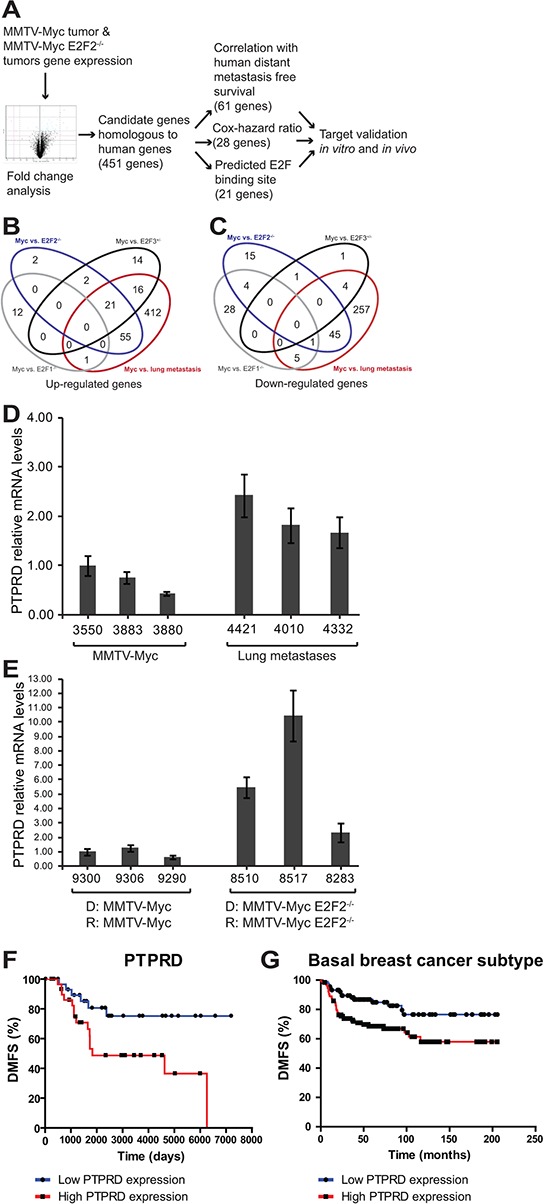
Pipeline for identification of genes regulating *E2F2*^−/−^ breast cancer metastasis To examine the regulatory mechanisms involved in *E2F2*-mediated human breast cancer metastasis, we established a pipeline for analysis. Genes that were differentially expressed by MMTV-*Myc*, MMTV-*Myc* x *E2F2*^−/−^ and lung metastasis samples **A–C.** were identified. Mouse gene expression data for significantly expressed genes was clustered together with human datasets, revealing 451 candidate genes that are homologous to human genes. We ranked these potential target genes based on their correlation with human distant metastasis free survival, their cox-hazard ratio, and the existence of E2F motifs proximal to the transcriptional start site. Comparison between MMTV-*Myc* tumors and lung metastases revealed elevated *PTPRD* expression in the lung metastases samples **D.** Comparison of MMTV-*Myc* WT21 non-metastatic tumors and MMTV-*Myc* WT21 *E2F2*^−/−^ metastatic tumors passaged in their respective genotype revealed increased *PTPRD* expression in the MMTV-*Myc* WT21 *E2F2*^−/−^ tumors (**E.** D = donor, R = recipient). Elevated levels of *PTPRD* were found to be correlated with human distant metastasis free survival (**F.** *p* = 0.0153) and is associated with basal breast cancer subtype (**G.** *p* = 0.0085).

In order to identify the genetic mechanism altered with the loss of *E2F2* that resulted in an increase of lung metastasis in *Myc* induced tumors we examined gene expression data from tumors and metastases. The goal of this analysis was to identify genes that were regulated directly or indirectly by *E2F2*. Importantly, we sought to examine genes that also affected human metastatic outcome. The data analysis pipeline (Figure 3A), began by examining fold change from MMTV-*Myc* and MMTV-*Myc E2F2*^−/−^ tumors as well as lung metastasis samples. 451 genes that are homologous to human were differentially expressed (Figure 3B and 3C). These putative target genes were then stratified based on their correlation to human distant metastasis survival, resulting in 61 genes with expression that correlated to human distant metastasis survival (Supplementary Table 1). A cox-hazard ratio analysis further narrowed this list to 28 genes (Supplementary Table 2). To then identify direct E2F target genes, we examined these genes for predicted E2F binding sites. This analysis predicted that 21 genes had E2F binding sites in a loose prediction and 3 genes in more stringent motif predictions (Supplementary Table 3). Combining these various criteria, we identified 7 genes that had potential to mediate metastasis by *E2F2* loss in *Myc* induced breast cancer. Of those 7 genes, *PTPRD* was the strongest candidate but appeared to be an indirect *E2F2* target. Interestingly, *PTPRD* was recently identified in the TCGA project [32] as being significantly mutated in breast cancer.

Microarray analyses revealed that *PTPRD* was upregulated in lung metastases samples compared to tumor samples. This finding was confirmed by qRT-PCR (Figure 3D). Furthermore, examination of MMTV-*Myc* WT21 tumors and MMTV-*Myc* WT21 *E2F2*^−/−^ tumors that were passaged in their respective background showed relatively high *PTPRD* expression in the metastatic MMTV-*Myc* WT21 *E2F2*^−/−^ tumors compared to the MMTV-*Myc* WT21 tumors, further confirming our initial findings (Figure 3E). The metastatic importance of *PTPRD* in human breast cancer was observed in the examination of the distant metastasis free survival (DMFS) curve where high levels of *PTPRD* were associated with poor DMFS outcomes (Figure 3F; *p* = 0.0153) relative to low levels of *PTPRD* and was associated with basal subtype of human breast cancer (Figure 3G; *p* = 0.0085, hazard ratio = 2.3 (1.2–3.78)).

### *E2F2* knockdown in human breast cancer increases migration and lung colonization

**Figure 4:**
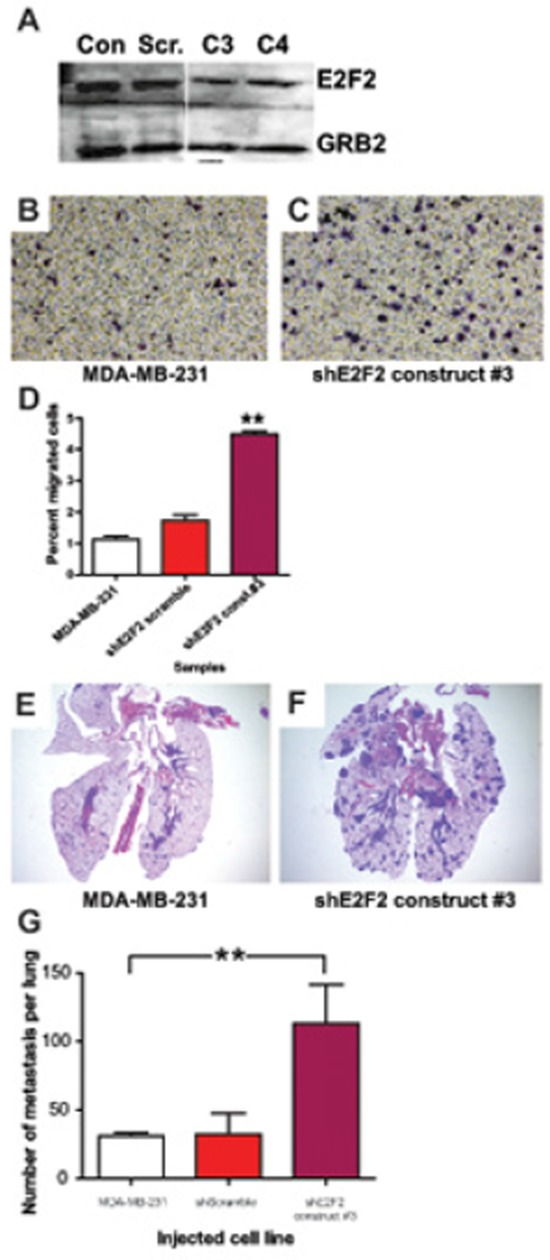
*E2F2* knockdown in human breast cancer increases migration and lung colonization *E2F2* knockdown in human breast cancer was achieved by transfection of MDA-MB-231 cells with sh*E2F2*. Efficacy of *E2F2* knockdown was assayed by western blotting **A.** with untransfected MDA-MB-231 (Con.), MDA-MB-231 transfected with shScramble (Scr.), sh*E2F2* construct #3 (C3), sh*E2F2* construct #4 (C4). Migration of MDA-MB-231 control cells **B.** and with *E2F2* knockdown **C.** in transwell migration assays revealed that the percentage of cells that migrated across the membrane increased when the level of *E2F2* was decreased (**D.** *p* < 0.0001). In colonization assays with and without the knockdown, lesions were found in the lungs of mice injected with MDA-MB-231 **E.** and greatly increased with transfection of sh*E2F2* **F.** Quantification of the numbers of metastatic lesions revealed an increased number of metastatic lesions in mice injected with MDA-MB-231 transfected with sh*E2F2* (**G.** *p* = 0.0184).

In order to establish a system where we could assess the effects of *PTPRD* knockdown, we began by examining whether the mouse effects of *E2F2* translated to human breast cancer. Using an shRNA approach we knocked down *E2F2* levels in MDA-MB-231 cells. The knockdown of *E2F2* was validated in stable clones through immunoblotting for *E2F2* in relation to the *Grb2* loading control (Figure 4A). No alterations to proliferation were noted (Supplementary Figures 1 and 2). Parental cells and a scrambled shRNA construct were used as controls when we assayed for migratory ability in a transwell assay. The transwell migration assay revealed that knockdown of *E2F2* resulted in increased cell migration through the transwell insert membrane relative to controls (Figure 4B–4D, *p* < 0.0001; Supplementary Figures 1 and 2; *p* < 0.05). This increased propensity to migrate was also demonstrated through a wound healing assay (data not shown).

Given that the *in vitro* experiments only examine a portion of the functions necessary for metastasis, we examined the ability of the cells to colonize the lungs by injecting the cells into the bloodstream. As expected, in control cell lines this resulted in localized discreet colonization (Figure 4E). Strikingly, knockdown of *E2F2* resulted in a significant increase in metastatic lesions (Figure 4F). Quantitation of these effects revealed a large increase in number of metastatic lesions in a section of the lung by more than four folds (Figure 4G, *p* = 0.0184). Taken together our results *in vitro* and *in vivo* showed that knockdown of *E2F2* increased the metastatic capability, correlating well with the increased metastasis in the MMTV-*Myc E2F2*^−/−^ mouse model.

### *PTPRD* knockdown in human breast cancer decreases migration and lung colonization

**Figure 5:**
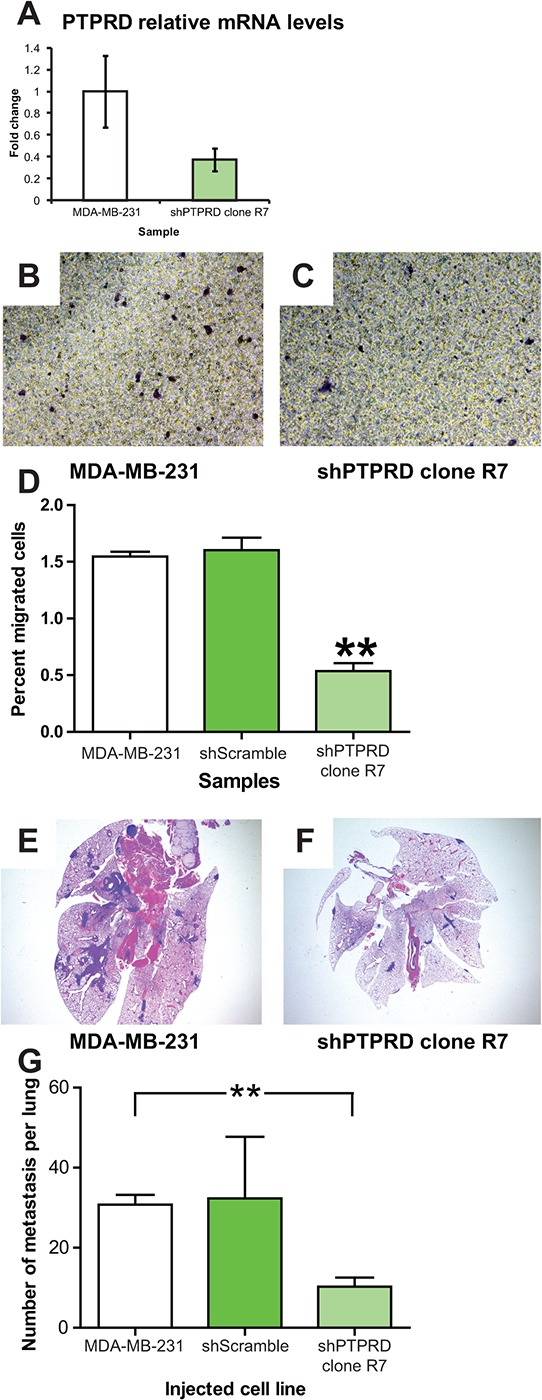
*PTPRD* knockdown in human breast cancer decreases migration and lung colonization *PTPRD* knockdown in human breast cancer was achieved by transfection of MDA-MB-231 cells with sh*PTPRD*. Efficacy of *PTPRD* knockdown was assayed by qRT-PCR (**A.** *p* = 0.01). Migration of MDA-MB-231 control cells **B.** and with *PTPRD* knockdown **C.** in transwell migration assays revealed that the percentage of cells that migrated across the membrane decreased when the level of *PTPRD* was decreased (**D.** *p* < 0.0001). In colonization assays with and without the knockdown, lesions were found in the lungs of mice injected with MDA-MB-231 **E.** and decreased with injection of MDA-MB-231 transfected with sh*PTPRD* **F.** Quantification of the numbers of metastatic lesions revealed a decreased number of lesion in mice injected with MDA-MB-231 transfected with sh*PTPRD* (**G.** *p* = 0.0009).

After establishing an experimental system whereby *E2F2* knockdown increased metastatic potential, we then examined if loss of *PTPRD* would reduce metastasis in the same system. To this end, we used an shRNA approach to knockdown levels of *PTPRD* to 40% of wild type levels (Figure 5A, *p* = 0.01). No effects on proliferation were noted (Supplementary Figure 2B).

Effects of *PTPRD* knockdown were asssayed *in vitro* by a transwell migration assay. This demonstrated that knockdown of *PTPRD* resulted in a significant decrease of the percentage of cells that migrated through the transwell insert membrane (Figure 5B–5D; *p* < 0.0001; Supplementary Figures 3 and 4; *p* < 0.01 and *p* < 0.05, respectively). *In vivo*, knockdown of *PTPRD* resulted in fewer metastatic lesions in the lung relative to controls after cells were injected retro-orbitally (Figure 5E–5G; *p* = 0.0009). This result reflected our gene expression finding in the MMTV-*Myc* mouse model where *PTPRD* was found to be upregulated by 2.83 fold in lung metastasis compared to MMTV-*Myc* tumor samples. This fold change difference was further validated by using qRT-PCR which showed that *PTPRD* RNA expression was increased by 2 fold in lung metastases samples compared to MMTV-*Myc* samples (Figure 3D). Taken together, these data indicated that *PTPRD* knockdown significantly decreased metastatic capability.

### Regulatory network analysis indirectly connected *E2F2* and *PTPRD*

**Figure 6:**
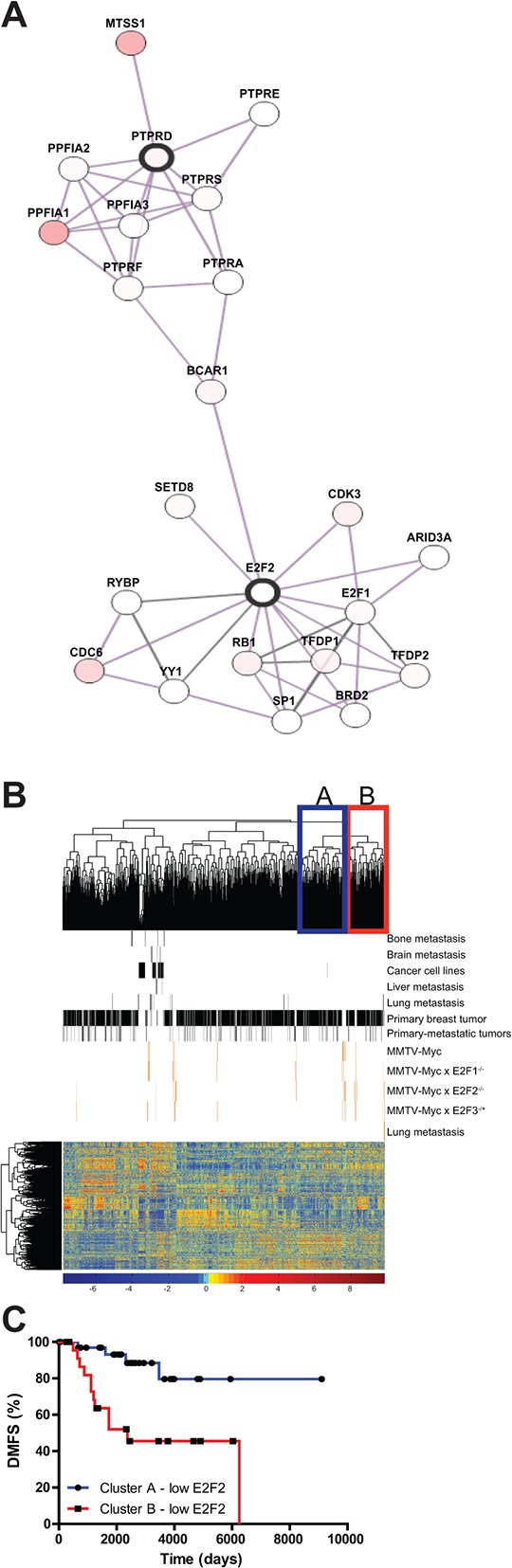
*E2F2*, *PTPRD*, and connections to human breast cancer We examined a protein-protein interaction network and found that *E2F2* and *PTPRD* were connected through *MYC* and *STAT3* **A.** Unsupervised clustering of human breast tumor datasets and mouse tumor dataset revealed a cluster in which lung metastasis samples and human tumors were clustered **B.** Stratification of human Distant Metastasis Free Survival data by *E2F2* pathway probability values showed differential effect of *E2F2* loss in human tumors **C.** *p*-value < 0.0001).

Examination of the networks linking *PTPRD* and *E2F2* showed that *E2F2* is potentially indirectly linked with *PTPRD* (Figure 6A). Given the role of *BCAR1* in mediating breast cancer invasion [36], it appears that there may be a critical link for mediation of metastasis in human breast cancer between *E2F2* and *PTPRD*. In order to compare the mouse model data with human breast cancer, we co-clustered the mouse tumors with human breast cancer samples. Interestingly, unsupervised hierarchical clustering revealed that the lung metastases all clustered together with a subset of human breast cancer samples (Figure 6B, cluster B). We predicted the probability of *E2F2* pathway activation in these subsets of human tumors and used it to stratify clinical gene expression samples. A comparison between cluster B and the closest neighboring cluster (Figure 6B, cluster A) for samples with low *E2F2* activity revealed low *E2F2* pathway activation in cluster B was correlated to decreased time to distant metastasis whereas in cluster A low probability of *E2F2* pathway activation was correlated to increased time to distant metastasis free survival (Figure 6C; *p* < 0.0001). Survival analysis showed that overexpression of *PTPRD* decreased time to distant metastasis in the basal subtype of human breast cancer (Figure 3G). In addition, examination of mouse gene expression data showed that in *PTPRD* was highly expressed in cluster B (Supplementary Tables 6 and 7; Supplementary Figure 5). Taken together these findings suggested that *E2F2* loss regulated breast cancer metastasis in a subpopulation of human tumors, potentially through a *PTPRD* signaling axis.

## DISCUSSION

Amplification of *Myc* has been associated with poor prognosis and distant metastasis in human breast cancer [37]. However, the MMTV-*Myc* transgenic mice are normally poorly metastatic, and only become metastatic with additional genetic events [17]. *Myc* function *in vitro* is reliant upon the E2Fs, a family of transcription factors essential for *Myc* mediated cell cycle progression and apoptosis. The E2Fs have been predicted and genetically demonstrated to be critical for *Myc* induced tumors. Interestingly, the genetic crosses to delete *E2F2* in *Myc* induced tumors have provided different results for tumor latency [28, 29]. These effects are likely due to promoter differences, background, differences in transgene integration and expression as well as developmental timing of transgene activity.

Here we noted that *E2F2* loss in *Myc* induced tumors significantly increased metastasis. This finding was made in two separate *Myc* transgenic lines and suggests that a critical function of *E2F2* in *Myc* tumors is in regulation of metastasis genes. Strikingly, these results are quite distinct from other mouse models of breast cancer. For instance, loss of *E2F2* in MMTV-Neu and MMTV-PyMT transgenics vastly reduced metastatic capacity [13, 27]. Together, these results indicate that there are oncogene specific genetic programs that involve differential signaling through the E2F transcription factors. Importantly, these differences may be regulated by which E2F is activated. Given that other E2Fs can compensate for loss of individual E2Fs [38], it is possible that loss of *E2F2* in the MMTV-*Myc* mouse model increased metastasis by increasing the activity of other E2Fs and thus promotes metastasis by differentially regulating other genes involved in metastatic progression [30, 31]. We noted also that the transplantation of MMTV-*Myc* WT21 *E2F2*^−/−^ tumors into the background of MMTV-*Myc* WT21 produced significantly more metastases compared to MMTV-*Myc* WT21 tumors transplanted into the same background. This suggested that metastases from MMTV-*Myc* WT21 *E2F2*^−/−^ was cell autonomous. We observed increased metastasis when MMTV-*Myc* WT21 was transplanted into MMTV-*Myc* WT21 background, a small effect likely due to stromal alteration associated with surgery procedure. Further analysis to explore the mechanisms by which E2Fs influenced metastasis in these models is warranted.

GSEA analyses of EMT and lung metastases gene expression clusters revealed the expected enrichment of *SMAD2, SMAD3*, and *SMAD4* regulated genes in the EMT cluster. Interestingly, when lung metastasis samples were compared to EMT histological type tumors, there was an enrichment of an invasive ovarian tumors geneset in the lung metastases. Given that pulmonary metastasis is frequently observed in ovarian cancer [39], this result suggests that there may potentially be a shared pathway between lung metastases and invasive ovarian tumors.

To elucidate the mechanism by which *E2F2* mediated breast cancer metastasis, a gene expression approach for target identification was coupled with human breast cancer distance metastasis free survival times. This identified *PTPRD* as one of the strongest potential regulators of metastasis in the E2F knockout samples. Both *in vitro* and *in vivo* tests revealed that *PTPRD* activity was required for migration and colonization by human breast cancer cells.

*PTPRD* is a member of the Protein Tyrosine Phosphatases (PTPs) that is involved in various biological processes in cancer [40]. Specifically, *PTPRD* has been shown as tumor suppressor in glioma [33, 41], liver, lung, head and neck, colorectal and melanoma [42]. In glioblastoma *PTPRD* was shown to be deleted through array comparative genomic hybridization and copy number analysis [33]. Further exploration into the mechanistic function of *PTPRD* in glioblastoma demonstrated that the loss of *PTPRD* led to the accumulation of active phospho-*STAT3* in a p16Ink4A^−/−^ mouse model [34]. In breast cancer, *PTPRD* was discovered through The Cancer Genome Atlas project to be a novel gene that was frequently mutated in addition to *PTPN22*, suggesting the emerging roles of protein tyrosine phosphatases in breast cancer associated biological processes [32]. Specifically in breast cancer, *PTPRD* was found to be hypermethylated in late-stage breast cancer [43]. Our data suggests an additional role for *PTPRD* in mediating breast cancer metastasis in conjunction with the loss of *E2F2*.

While *PTPRD* is not predicted to be a direct transcriptional target of *E2F2*, analysis of the TCGA data revealed that there was potentially connection at the level of protein regulation, through BCAR1. Alternatively, *PTPRD* may function through *STAT3* [34] given the *STAT3* and *Myc* relationship [44]. Indeed, perturbation to *STAT3* levels has been shown to alter the expression of the oncogene *c-Myc* [45–47]. Moreover, given that *Myc* interacts with *Rb/E2F* [48, 49] and there is a *c-Myc* binding site on the promoter of *PTPRD*, it is plausible that the loss of *E2F2* allowed increased expression of *PTPRD* mediated by c-*Myc*. This increase of *PTPRD* could then lead to increased lung metastasis, a role for *PTPRD* and *E2F2* that is unique to a subpopulation of breast cancer. Further analyses revealed that *PTPRD* overexpression was correlated to decreased time to metastasis in human basal subtype tumors. Taken together these results demonstrated that *E2F2* and *PTPRD* expression in *c-Myc* tumors could act as additional risk factors associated with metastasis. Finally, although *MYC* is known to drive proliferation, it can also suppress metastasis [50] and therefore drug treatment directed towards signaling molecules downstream of *MYC* signaling should avoid affecting *E2F2* signaling to maintain metastasis suppression.

## MATERIALS AND METHODS

### Animal work

Animal use and husbandry was in accordance to institution and federal guidelines. MMTV-*Myc* [14], MMTV-*Myc* (WT21) [26] and *E2F2*^−/−^ [51] mice were bred and genotyped as previously described [28]. MMTV-*Myc E2F2*^−/−^ females were kept in continuous breeding cycles. Mice were palpated weekly for tumor presence and tumors were measured by calipers weekly until endpoint when primary tumor reached 20 mm in the largest dimension [28]. Primary tumors were harvested for flash frozen samples, histological analysis and frozen viable transplantable tumors. Lungs were analyzed histologically for metastasis. Large metastatic lesions were flash frozen prior to RNA isolation by RNeasy according to the manufacturer's protocol (Qiagen, Valencia, CA). The quality of RNA samples were checked using Agilent Bioanalyzer (Agilent Biotechnologies, Santa Clara, CA) before being run on Affymetrix GeneChip Mouse Genome 430A 2.0 array chip (Affymetrix, Santa Clara, CA). Transplant assays were performed by transplanting 2.00 mm fragments of either MMTV-*Myc* WT21 or MMTV-*Myc* WT21 *E2F2*^−/−^ tumor into MMTV-*Myc* WT21 and MMTV-*Myc* WT21 *E2F2*^−/−^ backgrounds. 20 mice per genotype were used in the transplantation experiment and tumors were monitored as previously mentioned. Primary tumors and lungs were harvested and submitted for histological analyses as previously mentioned.

Colonization assays were performed by retroorbital injection of nude mice at a concentration of 5 × 10^5^ cells in 50 μl as previously described [52]. Mice observed for signs of labored breathing weekly for a minimum 30 days. At end point, mice were euthanized and lungs were harvested for routine staining. Quantification of colonization was achieved by quantifying the number of lesions found in the lungs.

### Gene analysis

Publicly available GEO datasets: GSE11121, GSE14020, GSE2034, GSE2603, GSE3494, GSE4922, GSE6532, GSE7390 and gene expression dataset from human breast cancer cell lines E-TABM-157 were obtained as well as their corresponding clinical annotations (Supplementary Tables 4 and 5). These gene expression datasets were pooled and normalized for batch effects by using Bayesian Factor Regression Modeling [53].

Validated training data from previous studies utilizing adenovirus infection of primary human mammary epithelial cells to build pathway signatures was used to identify samples with *Myc* and *E2F2* activation, *Myc* and *E2F2* signatures were applied as previously described to predict the pathway activation of *Myc* and *E2F2* [54–56]. Briefly, the normalized dataset was merged with training data and a binary regression algorithm was used to calculate the probability of pathway activation for *Myc* and *E2F2*.

Mouse gene expression dataset GEO24594 was merged with 6 lung metastasis samples (GEO71815). Briefly, CEL files were analyzed by Affymetrix Expression Console software to ensure that expressions were within bounds. Gene expression datasets were normalized using Microarray Suite 5.0 (MAS5) and Robust Multi-array Average (RMA) methods. Unsupervised hierarchical clustering was performed in RMA normalized samples with Cluster 3.0. Clusters characteristics were identified based on the majority of the histological subtypes present.

To identify samples with human relevance, gene expression from previously mentioned human tumors were co-clustered with mouse tumor dataset. Fold change difference (>1.5) between MMTV-*Myc*, MMTV-*Myc* x *E2F2*^−/−^ and lung metastasis samples were analyzed by GeneSpring. A *t*-Test was used to determine statistical significance for fold change analysis with *p*-value cut-off of 0.05. Fold change genes were examined for their correlation with human time to distant metastasis [57, 58] and Univariate Cox regression analysis, which allows genes to be ranked by effect size and does not require the normal assumption of proportional hazards to eliminate bias and maintain stability (Supplementary materials and methods) [59]. To determine whether candidate genes were in/direct E2F targets, genes were tested in GATHER [60] and SwissRegulon [61]. GSEA [62] analysis was completed with the Broad Institute GenePattern public server (http://www.broadinstitute.org/gsea/index.jsp). Regulatory network analysis was performed through examination of TCGA database [63, 64] using the genes: *E2F2* and *PTPRD*.

### Cell culture, transfection and migration assays

MCF7 and MDA-MB-231 were chosen based on the probability of *Myc* and *E2F2* pathway activation. Cells were cultured in DMEM, 10% Fetal Bovine Serum (FBS) and 2.0 mM L-glutamine.

shRNA constructs targeting *E2F2* and *PTPRD* were purchased from OriGENE (Rockville, MD). Cells were transfected using ExtremeGENE HP transfection reagent (ROCHE, Indianapolis, IN) according to manufacturer's protocol. Cells were selected using 2 μg/ml puro*Myc*in 48 hours after transfections. Both populations and colonies were tested using western blot to determine the knockdown efficiency. Wound healing assay and transwell migration assay was performed as previously described [65]. Transwell assays were quantified using ImageJ with Cellcounter plugin.

### qRT-PCR

RNA samples were isolated using RNeasy Plus mini-kit (Qiagen, Valencia, CA) according to the manufacturer's protocol. The primers for *PTPRD* (human) were F-TCACCAAGCTGCGTGAAATG and R-CAGCCATGGGATCTACAACAAA and the primers for *PTPRD* (mouse) were F-GGCTAGCCATCCTCCAATACC and R-TCCTGGGATTCCTCATATTCC (IDT, Coralville, IA). qRT-PCR was performed on 20 ng of total RNA (MDA-MB-231) or 200 ng of total RNA (MCF7) using QuantiTECT Sybr green PCR kit (Qiagen).

### Western blotting

Primary antibodies for immunoblotting were rabbit anti-*E2F2* (clone E-19 Santa Cruz, Dallas, TX, 1:300) or rabbit anti-*PTPRD* (Abcam, Cambridge, MA, 1:100).

### Data analysis

Statistical analysis was performed using GraphPad Prism 5 and GraphPad Quickcalcs. Non-parametric *t*-Test was performed on the quantification of tumor metastases, percent area reduction, and transwell migration assays. Differences in latency and distant metastasis free survival was examined by plotting Kaplan-Meier survival plot. To examine the percentage of mice with metastasis, Fisher's test with 2 × 2 contingency table was used.

## CONCLUSIONS

This study concludes that; 1. *E2F2* loss contributes to metastasis in *Myc* induced tumors; 2. *E2F2* may act through *PTPRD* to increase metastasis in the MMTV-*Myc* model of breast cancer.

## SUPPLEMENTARY DATA FIGURES AND TABLES
















