# Supplementary material for: Increased metastasis with loss of E2F2 in Myc-driven tumors
Source: Oncotarget. 2015 Oct 13;6(35):38210–24. doi: 10.18632/oncotarget.5690 (PMC4741994; doi:10.18632/oncotarget.5690)
Supplement: Supplementary file 1 [file oncotarget-06-38210-s001.pdf]

SUPPLEMENTARY DATA

R-code for Univariate concordance regression analysis is shown below. RMA normalized gene expression data, including their corresponding metastasis clinical annotation, in .CSV format was uploaded to R. The script was run through RStudio.

```
logistic = function(b)
{
name <- readline(prompt="c+' Filename: ")
name2 <- readline(prompt="Gene List Name: ")
tf <- readline(prompt="Trancription Factor: ")
print ("Input Transcription Factor Quantile")
m < -scan(n = 1)
print ("Quantile Cut-Off for Gene Selection")
u < -scan(n = 1)
library(concreg)
data=read.csv(file.choose())
temp=tempfile()
Level < -data[ , colnames(data)==tf]
if (b==0) {
E2F2Q < -ifelse(Level < =quantile(Level, m, na.rm=TRUE), 0, 1)
d2=cbind(data, E2F2Q)
n=ncol(d2)
d=d2[ ,-c(1,2,n)]
for (v in d) {
d4 < -cbind(v, d2)
fit < -concreg(Surv(TDMFS, EDMFS)~ v, data=subset(d4, E2F2Q==0), maxit = 1000)
c < -exp(coef(fit))/(1+exp(coef(fit)))
cat(.5+abs(.5-c), fit$prob, exp(coef(fit)), '\n', append=TRUE, file=temp)
}}
else {
E2F2Q < -ifelse(Level > =quantile(Level, 1-m, na.rm=TRUE), 1, 0)
d2=cbind(data, E2F2Q)
n=ncol(d2)
d=d2[ ,-c(1,2,n)]
for (v in d) {
d4 < -cbind(v, d2)
fit < -concreg(Surv(TDMFS, EDMFS)~ v, data=subset(d4, E2F2Q==1), maxit = 1000)
c < -exp(coef(fit))/(1+exp(coef(fit)))
cat(.5+abs(.5-c), fit$prob, exp(coef(fit)), '\n', append=TRUE, file=temp)
}}
j=read.table(temp)
rownames(j)=colnames(d)
colnames(j) < -c("c", "P-Value", "Hazard Ratio")
final=write.csv(j, file=name)
d < -read.csv(name)
d5=subset(d, c >=quantile(c, u))
d3=t(d5)
d4=d3[1, ]
x < -(length(d4)-1)
y < -length(d4)
for (i in 1:x)
{
f < -cat(d4[[i]], "+", '\n', append=TRUE, file=name2)
}
f < -cat(d4[[y]], append=TRUE, file=name2)
}
```

SUPPLEMENTARY METHOD

Gene expression datasets were used to build a human database for distant metastasis free survival (DMFS). Downloaded datasets were limited to Affymetrix U133A platform to minimize data loss due to differences in probes across multiple different platforms. Raw data (.CEL files) from 9 gene expression datasets were downloaded from Gene Expression Omnibus (GEO) database. Files were normalized using Robust Multi-array Average (RMA) and Microarray Suite 5.0 (MAS5) options in Affymetrix Expression Console.

Probe IDs between Affymetrix human U133A chip and Affymetrix mouse 430A 2.0 were compared by using ChipComparer (http://chipcomparer.genome.duke.edu/) to limit analyses only to gene expressions that were quantified both in human and mouse datasets. To eliminate batch effect, datasets were normalized using Bayesian Factor Regression Modeling (Carvalho, 2008). The resulting combined dataset was then filtered to eliminate genes with standard deviation <2 prior to performing unsupervised hierarchical clustering. Unsupervised hierarchical clustering resulted in 8 clusters.

To examine the differential effect of E2F2 pathway activation probabilities on DMFS, we predicted the probability of E2F2 pathway activation on all human breast cancer samples. z-score was calculated for 124 human breast cancer samples in cluster A and 99 human breast cancer samples contained in cluster B. Low E2F2 pathway activation in cluster A ($N = 34/124$) and low E2F2 pathway activation in cluster B ($N = 24/99$) was compared by means of Kaplan-Meier survival curve using GraphPad 5.0.

To examine the differential effect of PTPRD gene expression on cluster B, z-score was calculated for 99 human breast cancer samples contained in cluster B. High quartile (low PTPRD expression, $N = 32/99$) and low quartile (high PTPRD expression, $N = 31/99$) was compared by means of Kaplan-Meier survival curve using GraphPad 5.0.

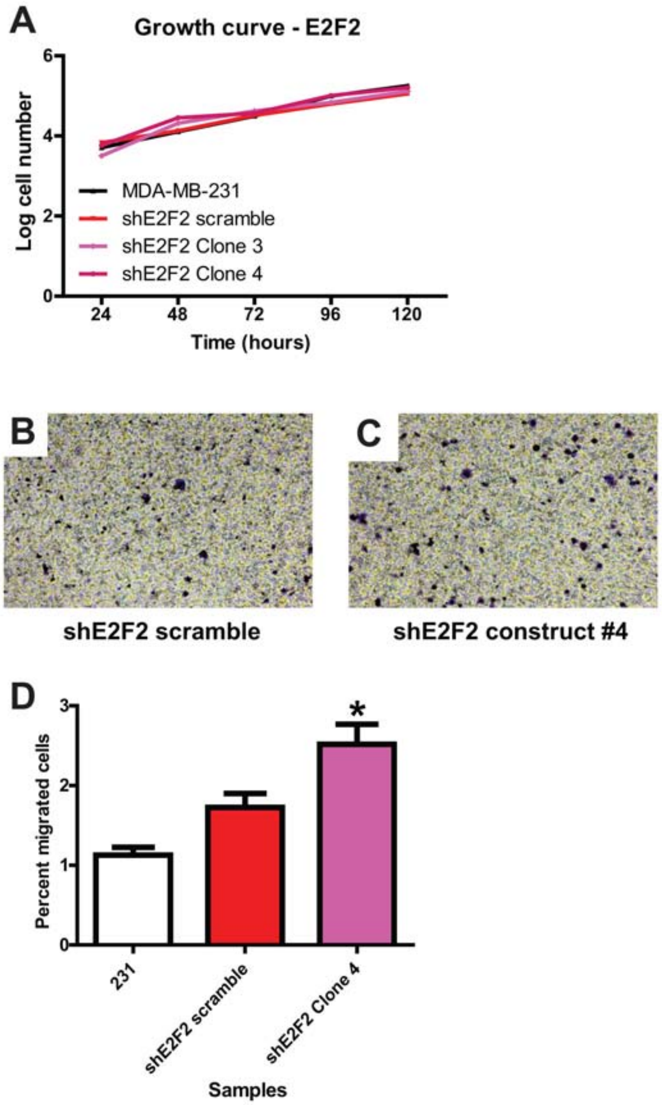

Supplementary Figure S1: Confirmation of the effects of E2F2 knockdown on MDA-MB-231 cell line.

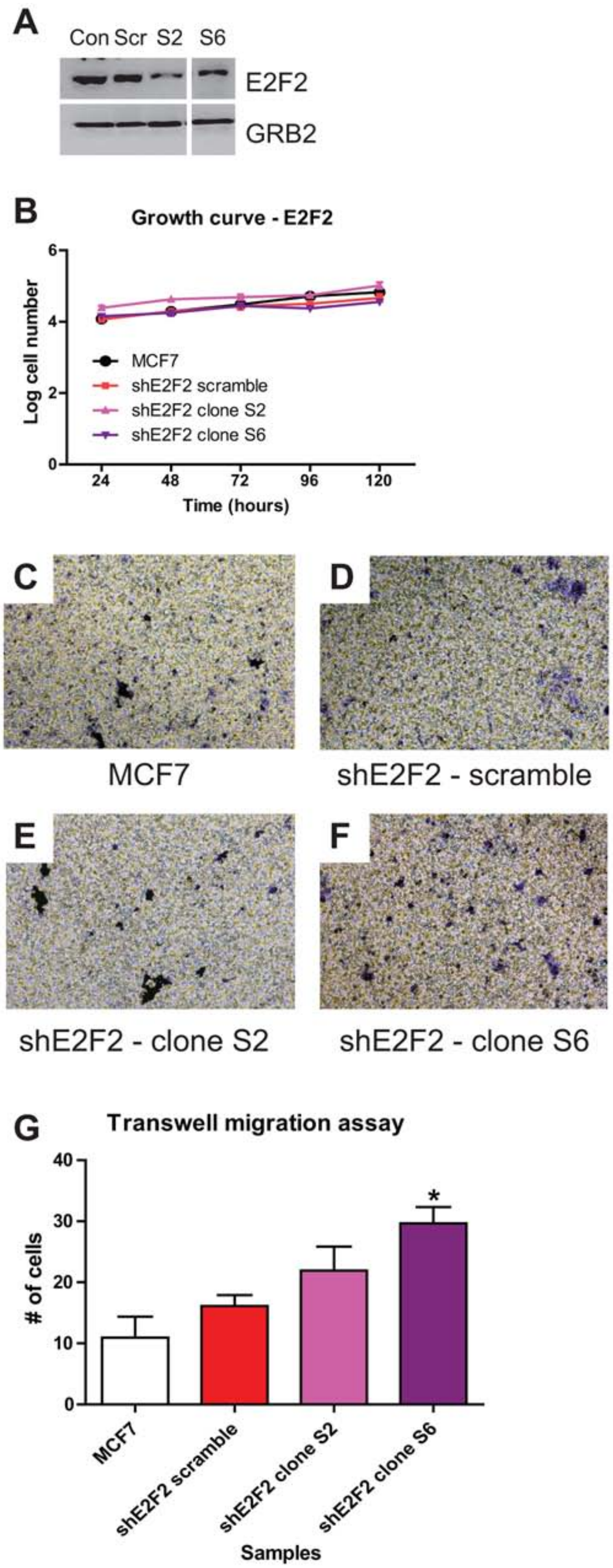

Supplementary Figure S2: E2F2 Knockdown in MCF7 cell line increased migration *in vitro*.

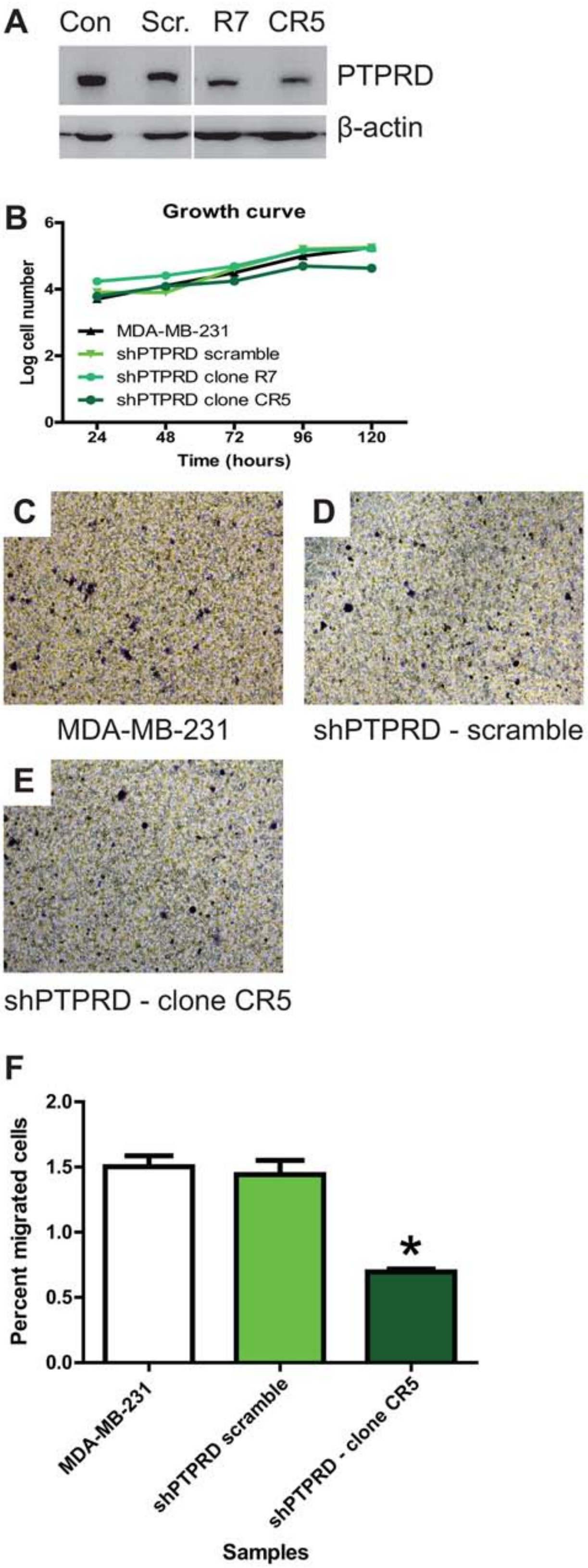

Supplementary Figure S3: Confirmation of the effects of PTPRD knockdown on MDA-MB-231 cell line.

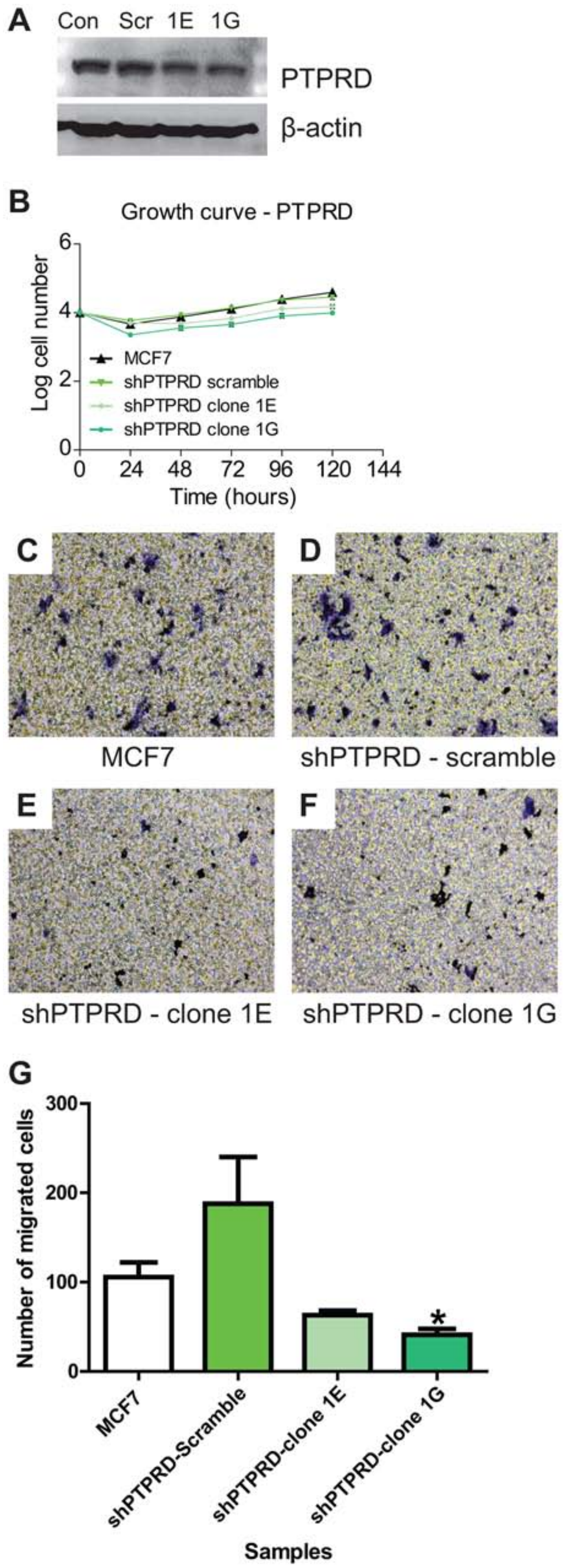

Supplementary Figure S4: PTPRD knockdown in MCF7 cell line decreased migration *in vitro*.

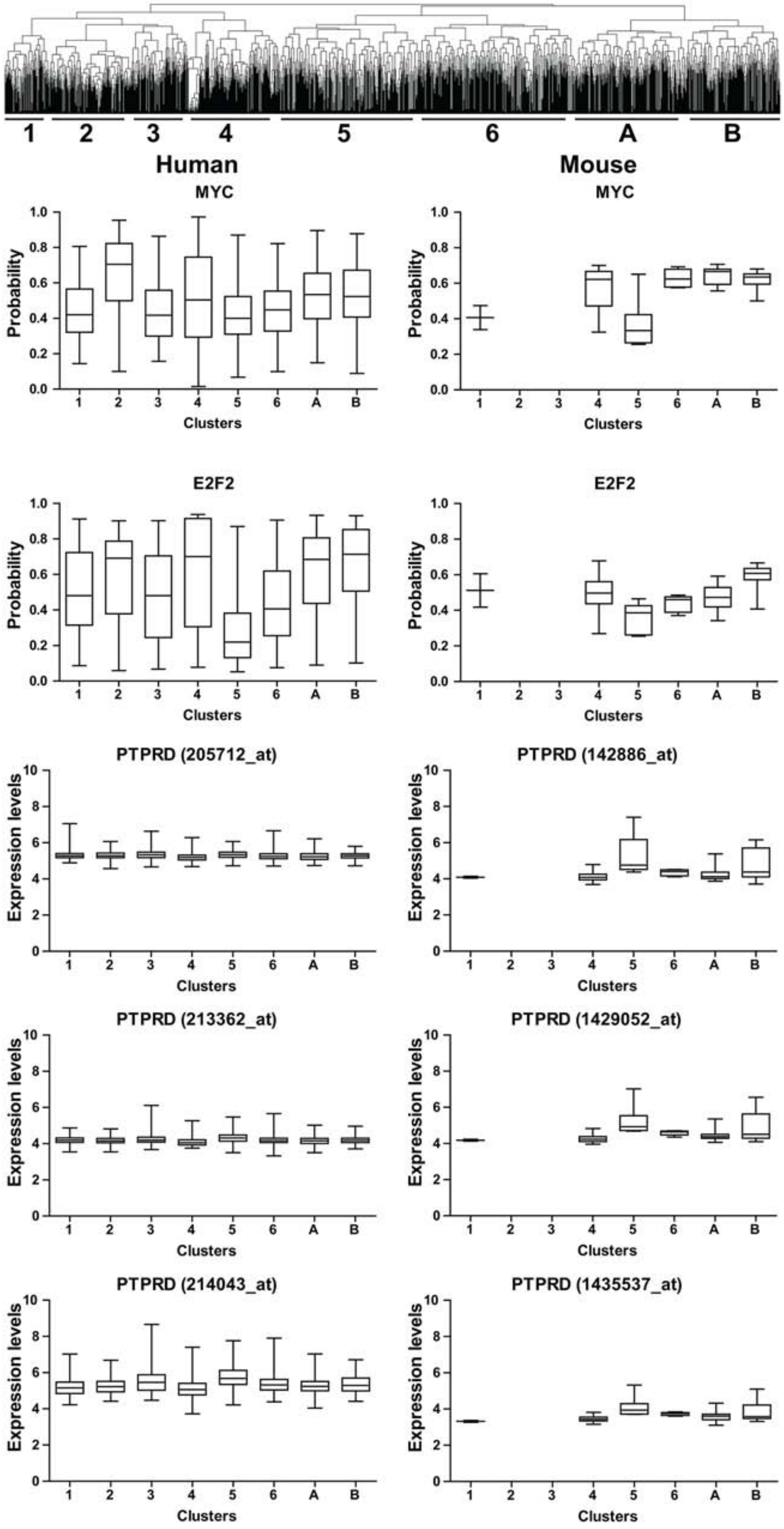

Supplementary Figure S5: Expression patterns of Myc, E2F2, and PTPRD in clusters previously identified by co-clustering of human and mouse gene expression datasets.

Supplementary Table S1: Homologous putative genes with relevance to DMFS.

Supplementary Table S2: Genes with significant Cox-hazard ratio.

Supplementary Table S3: Genes with putative E2F binding site.

Supplementary Table S4: Legend for mouse samples.

Supplementary Table S51: Legend for human samples.

Supplementary Table S6: Myc, E2F2, and PTPRD levels of human samples.

Supplementary Table S7: Myc, E2F2, and PTPRD levels of mouse samples.
